# Effect of air fryer frying temperature on the quality attributes of sturgeon steak and comparison of its performance with traditional deep fat frying

**DOI:** 10.1002/fsn3.2472

**Published:** 2022-01-20

**Authors:** Li Liu, Pan Huang, Wei Xie, Jinlin Wang, Yujin Li, Haiyan Wang, He Xu, Fan Bai, Xiaodong Zhou, Ruichang Gao, Yuanhui Zhao

**Affiliations:** ^1^ College of Food Science and Engineering Ocean University of China Qingdao Shandong Province China; ^2^ Hangzhou Qiandaohu Sturgeon Technology Co., Ltd. Hangzhou China; ^3^ Hisense (Shandong) Refrigerator Co., Ltd China; ^4^ Jiangsu Baoyuan Biotechnology Co., Ltd. Lianyungang China; ^5^ School of Food and Bioengineering Jiangsu University Zhenjiang China

**Keywords:** air‐frying, digestibility, flavor, quality, sturgeon

## Abstract

In this study, the influence of air‐frying temperature on the physical properties of sturgeon steaks was explored. Meanwhile, the comparison of traditional deep fat frying (TF) and air fryer frying (AFF) methods on the quality, flavor, and digestibility of sturgeon steaks were investigated. The results indicated that along with the increase of temperature (130, 160, and 190℃) for 15 min, the moisture content of AFF sturgeon steak surface decreased dramatically while that of interior was well preserved. The applied texture property analysis exhibited that AFF sturgeon steak showed the enhanced elasticity, low hardness, and soft texture. The results indicated that AFF sturgeon steak contained higher essential amino acid content than TF sturgeon steak. More flavor compounds (aldehydes, alcohols, and esters) were produced after AFF than TF. Although the digestibility of fried sturgeon steaks decreased after frying, AFF sturgeon steaks were digested rapidly in the stomach and intestine. Conclusively, AFF sturgeon steaks exhibited a crispy texture, appealing flavor, and low oil content. This work provides a certain reference for the suitable frying methods in the processing industry of sturgeon products.

## Graphical abstract

Under the optimum conditions, air‐fried sturgeon steak had crisp texture, attractive flavor, and low oil content, which can meet the preference of consumers. Compared with deep fat fried surimi, air frying can be considered as a healthy technique for preparing attractive fried food.

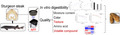

**Highlights**

- Sturgeon steak was process by air‐frying with minimum oil content in hot air.
- The influence of temperature of air‐frying and traditional deep fat frying was investigated.
- The air‐fried sturgeon steak was tender with a crispy crust by texture property analysis and moisture analysis.
- Air‐firied sturgeon steak has higher essential amino acid content than traditional firied sturgeon steak.
- Air‐firied sturgeon steak demonstrates relatively better flavor and digestibility.

## 1 INTRODUCTION

The *Acipenser*, also known as the sturgeon, has great biological and economic value in simplify processing owing to the lack of intramuscular spines. The sturgeon is a species of giant fish with high‐quality protein and a variety of vitamins and minerals (Boughattas et al., 2020). At present, the farming production of sturgeon in China accounts for more than 86% of the world (Wang et al., 2019). Caviar is the representative processed products of sturgeon, while boneless meat of sturgeon is less involved, causing a tremendous waste of sturgeon meat (Bronzi & Rosenthal, 2014). The essence of frying can be regarded as the process of dehydration which involves the transfer of heat and mass, resulting in a series of physical and chemical changes in food (Dueik & Bouchon, 2011). Deep‐fried fish products are very popular all over the world, not only for the characteristics of crisp and delicious, but also for the simple and convenient production process. However, excessive consumption of fried food can bring serious health risks, such as diabetes, cardiovascular disease, and obesity (Cahill et al., 2014; Hakami et al., 2013). Therefore, researchers pay more attention to the development of new low‐fat products to maintain the ideal flavor and texture of fried food.

Recently, there were new methods developed to lower the oil content of fried food, compared to microwave and low‐pressure frying, air‐frying is a better method in the maintenance of food quality and safety (Song et al., 2020). Air‐frying is a new technology of cooking fried food by spraying hot air around raw materials to promote the even contact between food and oil droplets in hot air. The process is carried out in an air‐fryer device which simulates the heat current in boiling oil to dehydrate the sturgeon steak product, crisp the outside, and cook the inside. It has been gradually accepted as a new frying method because of smaller capacity, shorter cooking time, and fewer calories (Fabre et al., 2018). It is worth noting that air‐frying imparts the similar characteristics of the traditional fried product with a substantially lower absorbed fat, so it could be used as a relatively healthy frying method (Tian et al., 2017). In addition, air‐frying has different effects on the color, texture, and flavor of food compared with traditional frying because of the oil oxidation (Joshy et al., 2020). However, there are few reports on the use of air‐frying in the processing of sturgeon meat. Therefore, it is necessary to compare the effects of traditional frying in a pan and air‐frying on the quality of sturgeon steaks.

The aim of this study was to evaluate the effect of operating temperature of air‐frying on the quality attributes and digestibility of sturgeon steaks. Meanwhile, the performance of traditional deep fat frying (TF) and air fryer frying (AFF) was compared. The results provide a theoretical basis for the frying method of sturgeon and promote the development of the sturgeon industry, supporting processing studies for developing healthy fried foods.

## 2 MATERIALS AND METHODS

### 2.1 sample preparation

Sturgeon was provided by Sturgeon Aquatic Food Technology Development Co., Ltd (Quzhou, Zhejiang, China) and stored in refrigerator at −4℃ for 1 hr, which was thawed in water and tap water, and then peeled. The sturgeon meat was cut into steak with weigh of about 160 g and thickness about 3 cm. Then, sturgeon steaks were cleaned and wiped to remove the water on surface.

### 2.2 Frying process

A household air‐fryer bought from Joyoung Co. Ltd. (Shandong, China) and fryer bought from SILEDE Co. Ltd. (Guangdong, China) was employed to fry the sturgeon steaks. Refined palm olein oil was used for TF and the oil was enough to eliminate any change of temperature upon the onset of frying. Specifically, the frying time of air and deep frying was controlled at 15 min, while the temperature was tested at 130℃, 160℃, and 190℃. After that, the sample was taken from the fryer and cooled to room temperature for further analysis.

### 2.3 Moisture Content

The moisture content in sturgeon steak samples was determined in triplets at 105℃ for 24 hrusing an electric thermostatic blast drying box (DHG‐9090A, Jinghong Co. Ltd., Shanghai, China). The weight of fried samples was performed by the electronic scale with a precision of 0.01 g (TP302N, Jinghai Co. Ltd., Shanghai, China).

### 2.4 Color

The color of sturgeon steaks was measured in triplicates with a chromometer (NR60CP, 3nh Co., Ltd, Shenzhen, China). Total color change (Δ*E*) is defined in the following equation.

(1) $$\Delta E={\left[{\left(L‐L_{0}\right)}^{2}+{\left(a‐a_{0}\right)}^{2}+{\left(b‐b_{0}\right)}^{2}\right]}^{1/2}$$

The parameters determined were degrees of lightness (L*), *L*_0_ is the lightness of the control, redness (a*), *a*_0_ is the redness of the control, and yellowness (b*), *b*_0_ is the yellowness of the control.

### 2.5 Texture analysis

The texture was determined by the physical property instrument (TMS‐PRO, FTC, USA) according to the previous method (Lee et al., 2018). Sturgeon steak samples were divided into 1.0 cm × 1.0 cm × 1.0 cm and produced by different frying methods. The probe type of P36/R was descented at 60 mm/min and returned at 100 mm/min, and the compression ratio was 40%. Six parallel experiments were set for each sample.

### 2.6 Scanning electron microscope (*SEM*)

*SEM* (JSM‐5800 LV, JEOL Corporation, Tokyo, Japan) was used to observe the microstructure representation of the sturgeon steak at 20 kV. The sample was fixed in glutaraldehyde solution and maintained at room temperature for 3 hr. Then, the sample was rinsed with distilled water and dehydrated in ethanol solution (50%, 70%, 80%, 90%, and 100%, v/v) for 15 min. Then, the dried samples were plated with gold for observation.

### 2.7 Determination of amino acids

The amino acid composition of sturgeon steak samples was determined by the amino acid analyzer (L‐8900, Hitachi, Tokyo, Japan) according to reported in previous research (Jo et al., 2018). The analytical column consisted of polystyrene cross‐linked by divinylbenzene (60 mm × 4.6 mm, 3 μm), with sulfone groups as the active exchange sites. The sturgeon sample (0.015 g) was suspended in HCl (6 mol/L), hydrolyzed at 110℃ for 24 hr, the enzymatic solution was cooled to room temperature. Then, the cooled enzymatic solution was concentrated at 55℃, and the concentrated solution was lyophilized. The dry hydrolysate was dissolved in 10 mL of 0.2 M sodium citrate solution and filtered through a 0.45 μm membrane. The amino acid composition of the hydrolyzed products was analyzed with C18 column. The essential amino acid score (EAAS) was calculated as follows.

(2) $$\text{EAAS}=\frac{\text{mg of EAA in}\;1\;\text{g protein of test samples}}{\text{mg of EAA in}\;1\;\text{g protein of FAO/WHO reference pattern}}\times 100$$

### 2.8 Determination of fatty acids

The acids measurement was estimated as reported (Li et al., 2015), lipids were extracted with chloroform/methanol (2:1) containing 0.005% butylated hydroxytoluene (antioxidant), and esterification was performed using 14% boron trifluoride‐methanol reagent. Agilent 6890 N (GC‐MS) was equipped with a flame ionization detector (FID) and polar capillary column (HP‐Innowax polyethylene glycol, 0.25 mm × 30 m × 0.25 μm). The carrier gas was helium with 1.0 ml/min, injection temperature was 200℃, and the detector temperature was 250℃. The initial temperature was 50℃, temperature from 50 to 200℃ at 10℃/min for 15 min, gradiently from 200℃ (30 min) to 240℃. The injection volume was 1 μL and segmentation ratio was 1:50. The fatty acid content was determined by comparing the area of various analyzed fatty acids with the area of a fixed concentration of internal standard.

### 2.9 Volatile components analysis

The volatile components of sturgeon steak were extracted by the headspace solid‐phase microextraction gas chromatography mass spectrometry (HS‐SPME‐GC/MS) with the HP‐5MS capillary column (30 m × 0.25 mm ×0.25 μm, Agilent Technologies Co. Ltd., State of Delaware, USA). Each sample (5.0 g crushed sturgeon) and 10 mL saturated sodium chloride solution in a headspace vial and incubated for 10 min at 80℃, followed by volatiles adsorption (60℃, 30 min) using a carboxen/divinylbenzene/ polydimethylsiloxane (Car/DVB/PDMS) fiber and volatiles desorption (250℃, 1 min) in injector. Helium (99.99%) was used as carrier gas at a flow rate of 1.0 mL/min. A gradient heating program was set as follows: the initial temperature was 40℃ for 3 min. Then, the temperature rose to 200℃ at a speed of 6℃/min and remained for 5 min. Next, the temperature rose to 250℃ at a speed of 10℃/min and remained for 5 min. The carrier gas flow was ultra‐purified helium at a constant flow rate of 1.0 mL/min, and the mode was non‐splitting. The ion source was EI with the ionization energy of 70 eV, the ion source temperature was 150℃, and the transmission line temperature was 250℃.

### 2.10 In vitro digestibility

The in vitro digestibility of the protein from different sturgeon steak samples was assessed using a digestion model as described (Wen et al., 2015). The sturgeon steak samples were dispersed in 33 mm glycine buffer at pH 1.8 (1 mg/mL), then digested with pepsin (5 U/mg protein) for 90 min at 37℃, and then digested with trypsin and α‐chymotrypsin (6.6 U and 0.33 U/mg protein, respectively) for 60 min. Absorbance was recorded at 280 nm to indicate the amount of hydrolyzed peptide during digestion. Sturgeon steak samples were measured by adding trichloroacetic acid (15% final concentration, w/v) at different time points. The supernatant was collected and the soluble protein content was determined by the biuret method. The digestibility was calculated using the following equation:

(3) $$\text{In vitro digestibility}=\frac{\text{Soluble protein content}}{\text{Total protein content}}$$

### 2.11 Statistical analysis

All values in this study are expressed as the mean ± *SD* of three measurements. Test results are expressed as mean ± standard deviation (SPSS Statistics 25 software, SPSS Inc., Chicago, IL). The smallest significant difference between treatments (*p* < .05) was accepted.

## 3 RESULTS AND DISCUSSION

### 3.1 Moisture content (MC)

**FIGURE 1:**
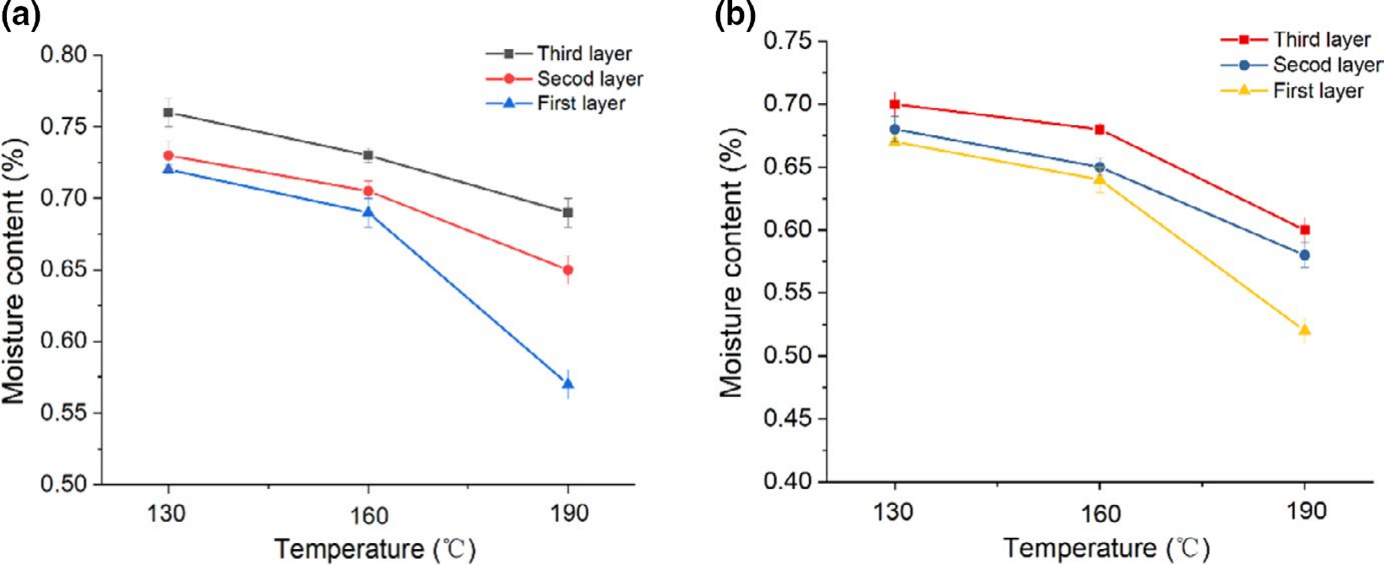
Moisture content of the first three layers of air fryer frying (a) and traditional deep fat frying sturgeon steaks under different frying time and temperature

Air fried products can attract consumers with crisp surface and proper moisture content inside. As shown in Figure 1a, the change of Moisture Content (MC) (as g/100 g dry matter) of different sturgeon steak samples was determined at different frying temperatures. As the temperature gradually increased, the MC of AFF sturgeon steak in the first layer decreased sharply from 0.723% to 0.571%. The variation trend of the second layer was relatively gentle and MC remained at 0.654% at 190℃. Strikingly, moisture could be well maintained in the third layer, and MC value can reach 0.690% after air‐frying at 190℃. TF sturgeon steak exhibited the same downward trend but the absolute value of MC was relatively low (Figure 1b), which was caused by the much faster heat transfer rate of liquid oil phase than of air phase, further affecting the moisture transfer (Teruel et al., 2015). In general, MC of fried sturgeon steaks by air has little change at 130℃, but the water loss rate is accelerated when the temperature exceeds 160℃. It was resulted that the surface temperature of the sturgeon steaks increased immediately and reached the boiling point quickly when the hot air began to blow, quickly forming a dried layer (Yang et al., 2019). Simultaneously, the drying layer can prevent further loss of internal moisture during frying. The results showed a violent moisture loss at any specific temperature above 160℃, which was in line with the characteristics of frying process (Ghaitaranpour et al., 2018).

### 3.2 Color

The browning reaction between reducing sugar and protein sources usually determined the color of the food surface (Zhang et al., 2020). The color of fried food affected its appearance and consumer acceptance. The lightness (L*), redness (a*), and yellowness (b*) were obtained by colorimeter to determine the surface color of fried sturgeon steak samples. The variation of L*, a*, and b* for sturgeon steak after two different fried methods were shown in Table 1. In the process of AFF, the L* decreased from 72.93 to 47.42 because of the moisture loss and the weakening of light reflection with the increase of temperature, resulting in the browning and darkening of sturgeon steak surface. The result indicated that the yellow and red deepened differently and the brightness decreased (Pedreschi et al., 2005). When the condition of AFF at 190℃ for 15 min, a* value changed to a positive value of 2.94, indicating the increase of redness. It is reported that high a* value is directly related to the production of carcinogenic compound acrylamide (Ataç Mogol & Gökmen, 2014). At 160℃ for 15 min, the L* of AFF sturgeon steak was significantly higher than TF, but a* and b* were lower (*p* < .05), which might result from redox reaction and browning reaction in TF. According to the calculation results of ΔE, the color difference of AFF sturgeon steak was less remarkable than TF sturgeon steak at 160℃ for 15 min, indicating that the method can keep the golden yellow color to a great extent.

**TABLE 1:** Effect of different frying methods on color indicators in sturgeon steak

Frying method		L*	a*	b*	∆*E*
Raw		72.93 ± 0.07^a^	0.71 ± 0.11^e^	2.76 ± 0.08^e^	0
TF	130℃	63.42 ± 0.08^b^	2.25 ± 0.16^c^	12.42 ± 0.03^c^	13.64 ± 0.07^d^
	160℃	49.65 ± 0.14^c^	12.33 ± 0.21^b^	15.25 ± 0.16^b^	28.86 ± 0.14^b^
	190℃	43.51 ± 0.20^d^	14.62 ± 0.15^a^	21.51 ± 0.08^a^	37.55 ± 0.16^a^
AFF	130℃	60.48 ± 0.18^b^	1.17 ± 0.06^d^	5.71 ± 0.13^de^	12.78 ± 0.13^d^
	160℃	54.91 ± 0.12^bc^	2.21 ± 0.08^c^	7.68 ± 0.06^d^	19.58 ± 0.10^c^
	190℃	47.42 ± 0.13 ^cd^	2.94 ± 0.12^c^	13.41 ± 0.14^bc^	27.73 ± 0.22^b^

Note

Superscript letters on the same column show the significant difference between the treatments (*p* < .05).

Data are expressed as means ± *SD*.

### 3.3 Analysis of texture characteristics of sturgeon steak

Texture property analysis is considered as an empirical test method to determine the texture properties of surimi products. It is usually used to simulate the chewing process of two extrusion deformation cycles. During the fried process of fish meat, myofibrillar protein and connective tissue are the two major factors that affect the changes in meat texture (Giri et al., 2019). The texture characteristics were shown in Table 2, The hardness of raw sturgeon steaks was 3.62 N, which was increased to 6.31 N after AFF at 130℃ for 15 min. The hardness of AFF sturgeon steaks increased further with the increase of air‐frying temperature, reaching the maximum value of 10.42 N in 15 min at 190℃. The moisture loss could lead to the increase of viscosity and mechanical energy of fried food, which caused the compact structure of fried food (Faisal et al., 2017). The elasticity of AFF sturgeon steaks grown along with the increase of temperature and reached the maximum value (8.92 mm) at 160℃. After that, when the temperature further climbed up to 190℃, the elasticity suddenly decreases. This tendency of elasticity may be attributed to the homogeneous and heterogeneous thermal denaturation of myosin and actin (Takahashi et al., 2005). Compared with AFF sturgeon steak, TF sturgeon steak had a significant improvement in chewability, while the other three texture characteristics had no significant difference. On the one hand, during the high temperature fried process, various covalent and non‐covalent bonded in the muscle tissue break, and the structure of the myofibrillar protein was changed, which might cause a loose structure. On the other hand, the high temperature of frying caused the continuous dissolution of connective tissue, resulting in the partial dissolution of collagen into gelatin or gel‐forming, while the longer time might lead to less connective tissue and looser texture in AFF processing (Tornberg, 2005). Therefore, texture analysis showed that AFF sturgeon steak at 160℃ had a softer and more crispy texture than TF sturgeon steak. According to the results of moisture content, color, and texture characteristics, it was necessary to further analyze the effect of different frying methods on fried sturgeon steak quality at 160℃ for 15 min.

**TABLE 2:** Texture profile analysis of sturgeon steak in different frying methods

Method		Hardness (*N*)	Elasticity (mm)	Chewability (mJ)	Adhesion (mJ)
Raw		3.62 ± 0.09^d^	7.46 ± 0.22^d^	17.0 ± 0.25^e^	1.35 ± 0.06^d^
TF	130℃	8.66 ± 0.08^bc^	13.42 ± 0.21^b^	44.36 ± 0.14^c^	6.17 ± 0.11^c^
	160℃	9.13 ± 0.10^b^	14.31 ± 0.18^a^	53.41 ± 0.38^b^	6.43 ± 0.15^bc^
	190℃	12.01 ± 0.05^a^	13.63 ± 0.08^b^	61.37 ± 0.16^a^	8.23 ± 0.23^a^
AFF	130℃	6.31 ± 0.17^a^	9.25 ± 0.23^c^	37.62 ± 0.33^d^	4.25 ± 0.21^ab^
	160℃	8.15 ± 0.06^c^	8.92 ± 0.06 cd	42.13 ± 0.21^c^	6.63 ± 0.08^b^
	190℃	10.42 ± 0.12^ab^	7.81 ± 0.14^d^	50.45 ± 0.18^b^	7.15 ± 0.14^ab^

Note

Different letters in each column indicate significant differences between groups (*p <*.*05*).

Abbreviations: AFF, air fryer frying; TF, traditional deep fat frying.

### 3.4 Fiber diameter and microstructure

**FIGURE 2:**
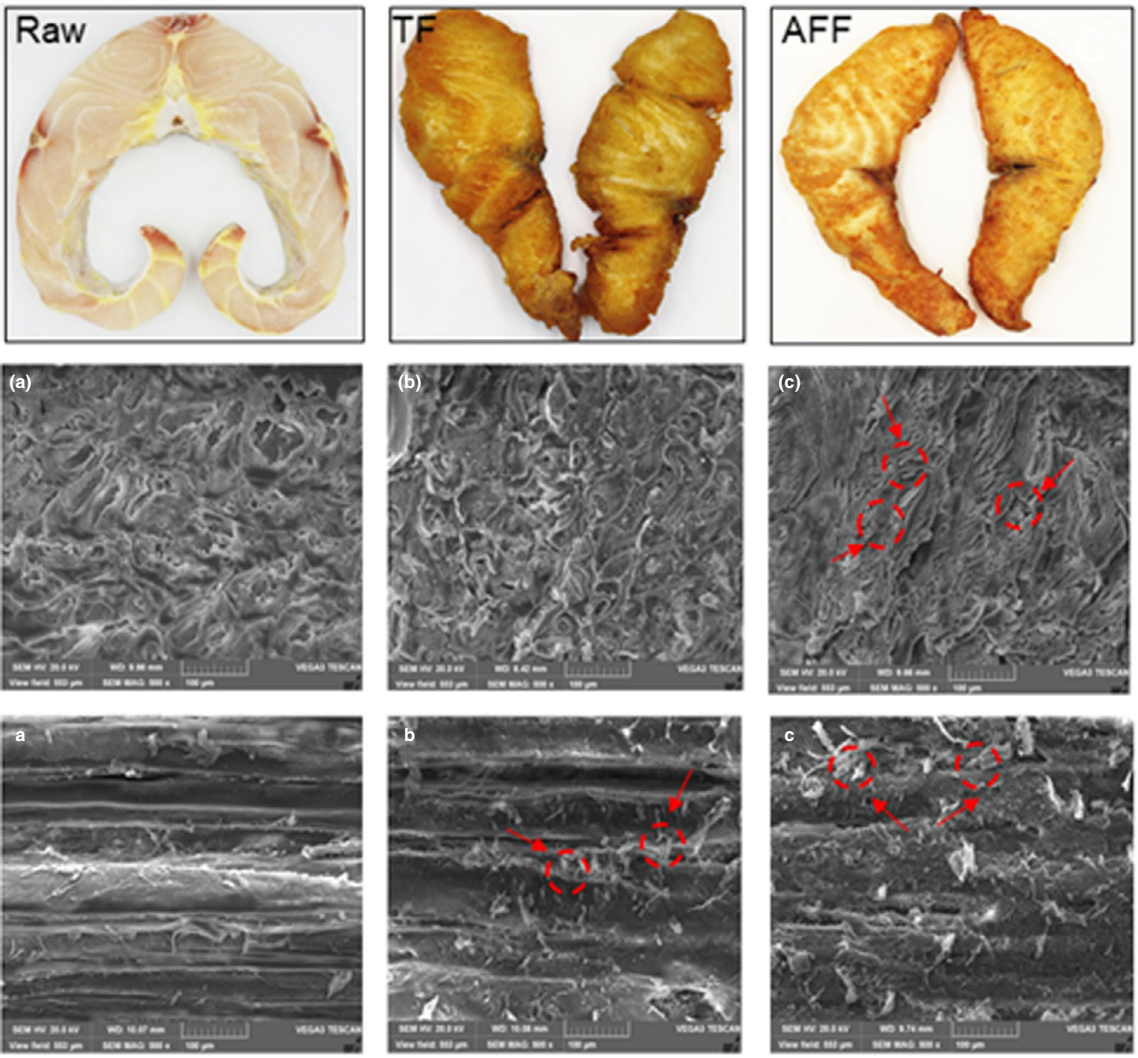
Electron micrograph of Raw (A, a), traditional deep fat frying (B, b), and air fryer frying (C, c) sturgeon steaks on the transverse (A, B, C) and vertical section (a, b, c) (500× magnification)

The transverse section of myofibrils of sturgeon steaks expanded and the gap increased during frying at 160℃ for 15 min (Figure 2a–c). The raw sturgeon steak structure was denser and the arrangement of collagen fibers were dense. In TF and AFF sturgeon steaks, the gap between the muscle fibers in the sturgeon steak sample was clearly visible, and the AFF treatment significantly increased the myofibril gap of the sturgeon steak. After frying, the protein denatures, and the drip loss originally contained in myofibrils overflow may lead to the increased gap between TF and AFF sturgeon steak (Roldán et al., 2013). Compared with TF sturgeon steak, AFF sturgeon steak had less open structure and rough structure, which might lead to softer texture, lower hardness, and shear force.

In the vertical section (Figure 2a–c), it can be observed that in the raw sturgeon steak, the fleshy structure was clear and smooth, and compactly arranged. Flocculent deposits were formed in the space between the muscle fibers in the fried sturgeon steaks. Furthermore, the flocculent structure of the AFF sturgeon steak was more disordered. This result indicated that the collagen of the muscle was thermally denatured after the frying. The fibroids of AFF sturgeon steaks had disordered arrangement, loose structure, distorted shape, and accompanied by obvious fracture because of higher temperature. Combined with the results of texture analysis of AFF sturgeon steak, the loose and disordered structure in *SEM* showed that AFF processing can give sturgeon products a crisp and fluffy taste.

### 3.5 Sturgeon steak amino acid composition analysis

Protein and amino acid content were important indicators to measure the nutritional value of food. As the main source of protein in food, fish had similar amino acid composition to the amino acid pattern required by humans (Oboh et al., 2016). As shown in Table 3, the 17 kinds of amino acids of fried sturgeon steaks were tested after different frying process at 160℃ for 15 min. All sturgeon steaks contained significant amounts of essential amino acids including lysine, leucine, threonine, and valine (Table 4). Almost all essential amino acid contents in the sturgeon steaks approached the Food & Agriculture Organization /World Health Organization (FAO/WHO) pattern, except for the phenylalanine and tyrosine, which were the significant components of aromatic amino acid. More remarkably, the sulfur‐containing amino acid content in the AFF sturgeon steak was nearly three times as high as that in the FAO/WHO pattern. The EAAS results suggested that AFF sturgeon steak had higher EAA values than TF sturgeon steak. The results demonstrated that frying could increase the amino acid content of sturgeon steak. Notably, AFF sturgeon steaks were composed of high‐quality dietary proteins that contain high amounts of almost all essential amino acids.

**TABLE 3:** Amino acid composition of sturgeon steak in different frying methods (g/100 g)

Amino acid	Raw	TF	AFF
His	0.59 ± 0.04^b^	0.91 ± 0.02^a^	0.95 ± 0.06^a^
Ile	0.73 ± 0.12^b^	1.24 ± 0.06^a^	1.27 ± 0.08^a^
Leu	1.3 ± 0.63^b^	2.3 ± 0.84^a^	2.18 ± 0.27^a^
Lys	1.57 ± 0.42^c^	2.57 ± 0.81^b^	2.81 ± 0.36^a^
Met	0.10 ± 0.04^b^	0.16 ± 0.06^b^	0.32 ± 0.01^a^
Phe	0.67 ± 0.06^b^	1.11 ± 0.04^a^	1.16 ± 0.13^a^
Thr	0.65 ± 0.08^b^	1.15 ± 0.05^a^	1.27 ± 0.20^a^
Val	0.74 ± 0.15^c^	1.27 ± 0.63^b^	1.43 ± 0.38^a^
∑EAA	6.35 ± 1.53^b^	10.71 ± 2.02^a^	11.39 ± 1.45^a^
Asp	1.68 ± 0.46^b^	2.71 ± 0.83^a^	2.8 ± 0.62^a^
Ser	0.70 ± 0.08^b^	1.22 ± 0.15^a^	1.22 ± 0.06^a^
Gln	2.77 ± 0.72^b^	4.75 ± 0.64^a^	4.85 ± 0.57^a^
Gly	0.74 ± 0.10^b^	1.56 ± 0.08^a^	1.52 ± 0.05^a^
Ala	0.92 ± 0.21^b^	1.73 ± 0.17^a^	1.75 ± 0.12^a^
Cys	0.22 ± 0.03^c^	0.43 ± 0.06^b^	0.67 ± 0.05^a^
Tyr	0.54 ± 0.06^c^	0.87 ± 0.05^b^	0.96 ± 0.08^a^
Arg	0.98 ± 0.08^b^	1.74 ± 0.06^a^	1.85 ± 0.06^a^
Pro	0.52 ± 0.04^b^	1.12 ± 0.06^a^	1.02 ± 0.02^a^
TAA	15.42 ± 2.82^c^	26.84 ± 1.93^b^	28.03 ± 2.45^a^
∑EAA/TAA (%)	41.18 ± 2.92^a^	39.90 ± 1.83^a^	40.64 ± 2.07^a^

Note

Superscript letters on the same column show the significant difference between the treatments (*p* < .05).

EAA and TAA represent essential amino acids and total amino acids, respectively.

Abbreviations: AFF, air fryer frying; TF, traditional deep fat frying.

**TABLE 4:** Essential amino acids score (EAAS) of sturgeon steak in different frying methods (mg/g protein)

Essential amino acids	FAO	Raw	TF	AFF
Ile	40	38.40	61.25	60.43
Leu	70	39.07	64.92	59.27
Lys	55	60.06	92.33	97.24
Met + Cys	35	72.74	111.78	115.29
Phe + Tyr	60	11.22	19.43	31.40
Thr	40	34.19	56.81	60.43
Val	50	31.14	50.19	54.43

Abbreviations: AFF, air fryer frying; TF, traditional deep fat frying.

### 3.6 Fatty acid component analysis

**FIGURE 3:**
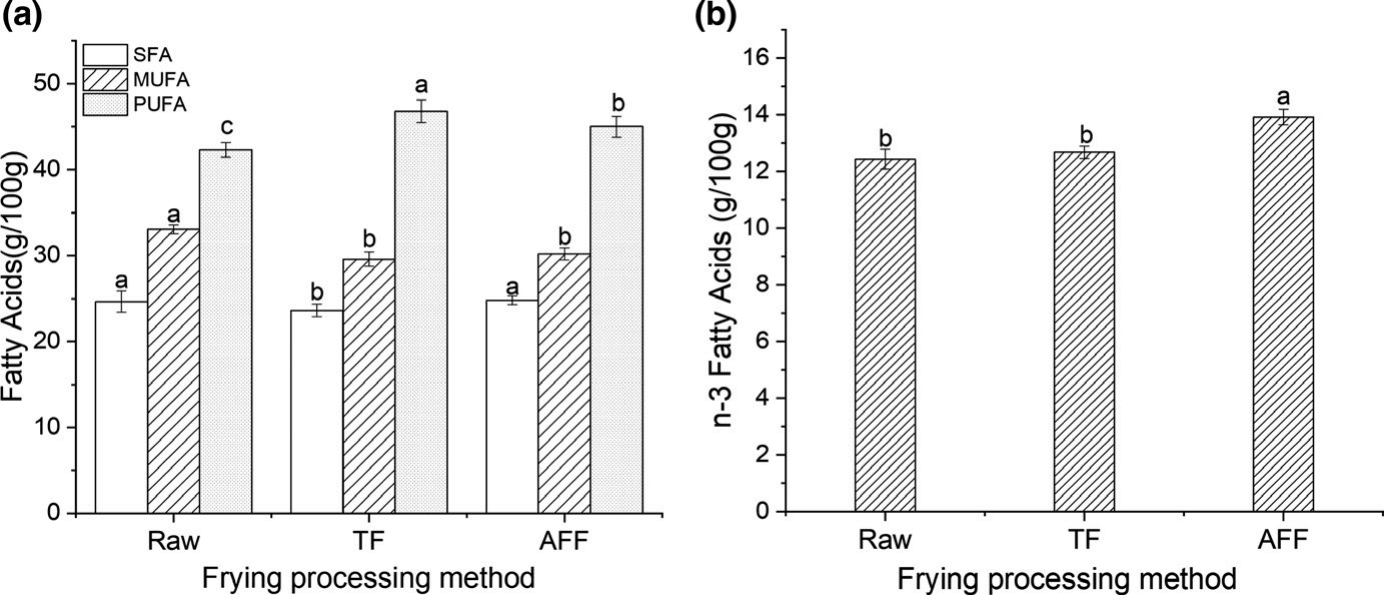
Fatty acid content by unsaturation (a) and total n‐3 fatty acids (the sum of ALA, EPA, and DHA) of different fried sturgeon steaks (b). SFA, saturated fatty acids; MUFA, monounsaturated fatty acids; PUFA, polyunsaturated fatty acids; Abbreviations: ALA, alpha linolenic acid; EPA, eicosapentaenoic acid; DHA, docosahexaenoic acid

Fatty acids component was important to evaluate the nutritional quality of fish products, some fatty acids with aroma can affect reactions to other flavors (Guy & Nottingham, 2014). As a result of the exchange and diffusion of fatty acids between muscle fat and cooking oil during lipid oxidation, the relative content of some fatty acids was changed (Zeng et al., 2016). As shown in Figure 3a, after frying at 160℃ for 15 min, the MUFA contents decreased, TF sturgeon steak had the highest content of PUFA. It might result that during the fried processing, the fat of sturgeon steak was exchanged with cooking oil (vegetable oil) containing a large amount of PUFA (Supawong et al., 2018). The temperature and time of frying also affected the exchange of fat between meat and cooking oil, leading to the change of fatty acids in fried food (Miranda et al., 2010).

n‐3 fatty acids are obtained from fish, fish oil, flaxseed, eggs, and dairy products, which has exhibited many health benefits (Riediger et al., 2009). As shown in Figures 3b, n‐3 fatty acids significantly increased (*p* < 0.05) during frying and the highest content was in AFF sturgeon steak. It was caused by the weak oxidation reaction in AFF but the nutritional value and texture characteristics of sturgeon steak were less affected (Marasca et al., 2016). Furthermore, the low degree of lipid oxidation in AFF processing resulted in less damage to nutritional value (Dhanapal et al., 2012). These results suggested that AFF sturgeon steak might be a good source of n‐3 fatty acids.

### 3.7 Volatile components analysis

**FIGURE 4:**
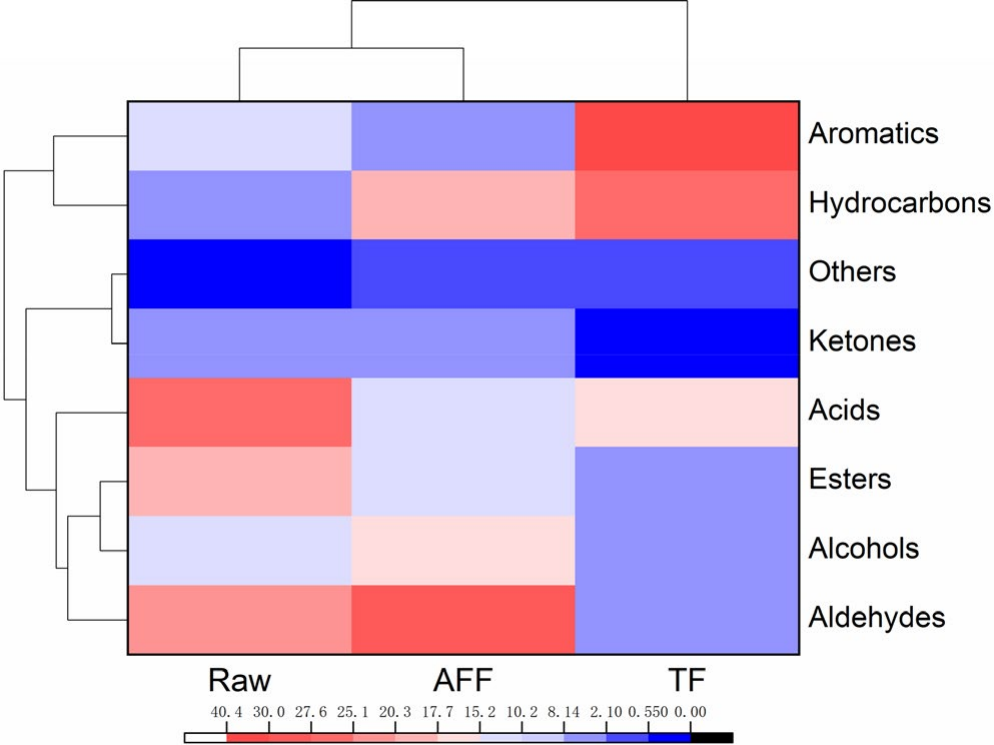
The hierarchical cluster heatmap of volatile components in different fried sturgeon steaks

The hierarchical cluster analysis of volatile compound of different fried sturgeon steaks at 160℃ for 15 min were shown in Figure 4. The volatile components in raw sturgeon steak were mainly aldehydes, acids, and esters. After frying, the content of aldehydes, hydrocarbons, esters, and aromatic compounds changed in different degrees, while the acids were decreased. This result showed that different frying methods changed the composition of the volatile components of the sturgeon steaks to some extent. Compared with raw sturgeon steaks, the aldehydes, alcohols, and hydrocarbons of AFF apparently increased, while the acids and aromatics significantly reduced. However, for TF sturgeon steaks, the volatile components were mainly hydrocarbons, acids, and aromatics. It showed that the composition of the volatile components of the sturgeon steaks during frying had changed. These volatile components were mainly aldehydes, alcohols, hydrocarbons, esters, and acids. And most of the compounds have been observed in several fish species (Iglesias & Medina, 2008).

The volatile components and relative contents of different fried sturgeon steaks were shown in Table 5. In the raw, TF, and AFF groups, 41, 43, and 41 volatile components were detected, respectively. The types of volatile components detected in the TF sturgeon steaks were significantly less than that in the AFF sturgeon steaks. This might result from the intense oxidation and Maillard reaction during the AFF, which accelerated the degradation of macromolecules.

**TABLE 5:** The volatile components and relative contents of sturgeon steak in different frying methods

	Raw	TF	AFF
Aldehydes			
2,3‐dimethyl‐pentanal	1.72	nd	nd
Benzaldehyde	8.68	nd	3.44
3‐hydroxy‐4‐methoxybenzaldehyde	5.38	nd	nd
Pentanal	1.46	0.37	2.99
Hexanal	5.49	5.06	11.51
Tetradecanal	0.38	nd	1.12
2‐methyl‐butanal	nd	0.38	1.72
2‐methyl‐propanal	nd	nd	1.03
3‐methyl‐butanal	nd	nd	2.28
Octanal	nd	nd	0.71
3‐hydroxy‐4‐methoxybenzaldehyde	nd	nd	3.25
Ketones			
Acetone	0.25	nd	nd
2,3‐butanedione	0.23	nd	nd
2,3‐pentanedione	0.72	nd	nd
3‐hydroxy‐2‐butanone	1.02	nd	nd
2‐butanone	nd	0.21	0.39
2‐heptanone	nd	nd	3.64
Alcohols			
3‐methy‐1‐butanol	6.01	0.66	6.88
1‐penten‐3‐ol	1.35	1.31	0.57
1‐propen‐2‐ol	nd	1.35	1.47
2‐penten‐1‐ol	0.46	nd	nd
2‐ethyl‐1‐hexanol	2.91	1.85	1.81
1‐pctanol	0.42	nd	0.76
Ethanol	0.59	nd	nd
1‐octen‐3‐ol	nd	nd	3.50
Benzyl alcohol	nd	nd	0.63
Hydrocarbons			
1,1‐diethoxy‐ethane	2.80	nd	nd
Trichloromethane	1.13	nd	4.80
3,5,5‐trimethyl‐2‐hexene	0.72	nd	nd
Cyclopropene	0.54	nd	nd
Pentadecane	0.51	0.46	4.42
Octane	nd	0.74	nd
6‐methyl‐undecane	nd	1.55	nd
Propane	nd	0.74	nd
Undecane	nd	3.20	2.36
3,5‐octadiyne	nd	2.21	nd
Hexadecane	nd	1.04	nd
Dodecane	nd	13.18	4.96
2‐Hexene	nd	0.60	0.61
3‐butyl‐2,4‐pentanedione	nd	0.50	0.59
Butylated bydroxytoluene	nd	0.51	nd
1R‐alpha‐pinene	nd	0.91	1.19
Acids			
*N*'‐isopropylureidoacetic acid	0.36	1.80	1.93
Malonic acid	5.34	nd	2.89
Acetic acid	0.86	0.60	0.55
Diethyl‐acetic acid	1.28	nd	0.95
4‐hydroxy‐butanoic acid	3.02	nd	0.81
Propanoic acid	15.22	0.85	1.13
Hexanoic acid	0.90	nd	2.28
1,2‐Benzenedicarboxylic acid	0.57	nd	nd
Malonic acid	nd	12.83	nd
4‐hydroxy‐butanoic acid	nd	0.34	nd
Nonanoic acid	nd	0.17	0.20
Esters			
Acetic acid, butyl ester	5.33	0.32	3.48
Tetraethyl silicate	3.01	0.25	1.07
Formic acid, hexyl ester	0.69	0.13	0.61
Tributyl phosphate	3.37	nd	nd
Diethyl phthalate	3.13	0.69	1.30
Dibutyl phthalate	0.63	nd	nd
2‐propanoic acid, 2‐ methyl ester	0.34	nd	nd
Propanetriyl ester	2.00	nd	2.09
Ethyl acetate	nd	0.55	nd
Methoxyacetic acid, benzyl ester	nd	2.88	3.71
Propanetriyl ester	0.23	0.42	2.09
Aromatics			
Toluene	1.01	1.98	1.03
Ethylbenzene	8.02	16.82	3.95
1,3‐dimethyl‐benzene	1.93	18.35	nd
1,3,5‐trimethyl‐benzene	nd	0.20	nd
1,2‐dichloro‐3‐methyl‐benzene	nd	2.95	2.65
Others			
Pyrazine	nd	1.04	0.66

Abbreviations: AFF, air fryer frying; TF, traditional deep fat frying.

Aldehydes are mainly produced by the oxidation of polyunsaturated fatty acids and have an overwhelming effect on the overall aroma (Zhou et al., 2016). The main aldehydes detected were hexanal, valeraldehyde, benzaldehyde, octanal, and tetradecane. These compounds were significantly higher in raw and AFF sturgeon steak than that of TF sturgeon steak. Hexanal had green grass and apple smell; octanal had fruit flavor; benzaldehyde had a pleasant nut fragrance; tetradecane had fish aroma. The results showed that these compounds may be the main reason for the mild and pleasant smell of AFF sturgeon steak.

Alcohol compounds are also one of the main volatile components in sturgeons, the threshold of unsaturated alcohol (e.g., 1‐octene‐3‐alcohol) is relatively low with a mushroom aroma and a similar metal flavor, which has a certain effect on the sturgeon flavor (Liu et al., 2016). The pleasant aroma of fried sturgeon steak might be caused by the higher content of 1‐octene‐3‐ol and 3‐methyl‐1‐butanol in AFF sturgeon steak.

Lipid oxidation is the main factor for the formation of esters in fried products, which contributes greatly to the flavor of sturgeon grilled products (Zhang et al., 2019). A total of six esters were detected in the fried sturgeon steaks. It was noteworthy that the content of acetic acid, butyl ester in AFF was more than TF sturgeon steak and close to that of raw sturgeon pickling, which had pleasant fruit flavor. Besides, ethyl acetate was only detected in TF sturgeon steak, which might due to the intense oil exchange during TF processing.

Hydrocarbons was another kind of abundant compound discovered in the sturgeon, which was reported to be derived from alkyl groups by lipid oxidation processes (Fu et al., 2009).

Among the aromatic compounds, toluene, 1,3‐dimethyl‐benzene, about four times as much ethylbenzene as the AFF sturgeon steak were detected in TF sturgeon steak. Toluene concentration of toluene had serious damage to the central nervous system of the human body. Furthermore, benzene was also involved in metabolic function, indicating insulin resistance, toluene could result in the birth defect (Bolden et al., 2015). The results indicated that AFF was healthier fried processing than TF (Poinot et al., 2014; Saillenfait et al., 2003).

Considering the results of volatile components, AFF sturgeon steak produced a lot of pleasant aromas and few carcinogens. Therefore, AFF was a more healthy and delicious processing method than TF for sturgeon steak products.

### 3.8 In vitro digestibility

**FIGURE 5:**
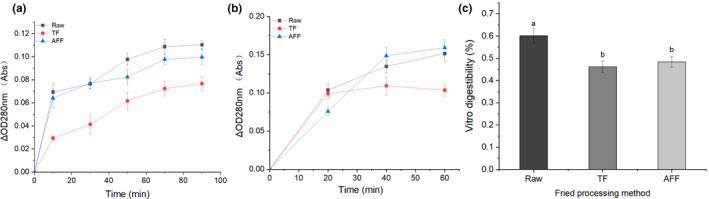
Digestive product absorbance of different fried sturgeon steaks at 280 nm treated by gastric pepsin (a) and pancreatic trypsin + α‐chymotrypsin (b) and in vitro digestibility (c)

In vitro digestion model is a common tool for evaluating the bioavailability of dietary proteins. By digesting with pepsin and trypsin, the content of soluble amino acids and peptides is measured, and the degree of protein decomposition is determined to determine the digestibility (Pennings et al., 2013). As shown in Figure 5, for all sturgeon steak, soluble amino acids and peptides increased rapidly in the first 60 min, and then tended to be stable (Figure 5a), indicating that the protein of sturgeon steak was mainly digested in the first hour. The content of digestive products in raw sturgeon steak was the highest, the digestive products content of AFF sturgeon steak was higher than TF. The results showed that the digestion rate of AFF sturgeon steak by pepsin was faster than that of TF. The high temperature of fried process gave rise to protein oxidation and denaturation, leading to protein aggregation and lower rate of digestion for fried sturgeon steak. Pepsin activity had a direct and quantitative relationship between protein carbonylation and aggregation induced and carbonyl derivatives by cooking (Sante‐Lhoutellier et al., 2008). Interestingly, the digestion rate of AFF sturgeon steak by pepsin was faster than that of TF, which resulted from the high content of Tyr, Phe, Leu, and Arg produced by the continuous digestion of aggregation in AFF sturgeon steak, increasing the opportunities for protease to contact recognition sites to form peptides.

After digestion by trypsin and α‐chymotrypsin, the content of digested products of AFF sturgeon steak was the highest (Figure 5b), and that of TF was the lowest. The results showed that trypsin and α‐chymotrypsin could hydrolyze AFF processed sturgeon steak easily. Moreover, digestibility decreased after fried processing (*p* < .05) and there was no significant difference between TF and AFF sturgeon steaks (Figure 5c). The digestion process of pepsin and trypsin was an orderly process, which had many effects on the digestibility of protein (Jiang et al., 2019). It was stemmed from the oxidation and carbonylation of proteins after frying, oxidized protein further caused the aggregation and settlement of proteins. The restriction sites of pepsin and trypsin were hidden, resulting in the change of the number of restricted sites and the decrease of digestibility (Du et al., 2018). In addition, pepsin and trypsin usually hydrolyze tyrosine, phenylalanine, aspartic acid, glutamic acid, methionine, leucine, and tryptophan at the carboxyl end of the protein. The higher the content of these amino acids, the greater the probability of enzymatic hydrolysis, which was consistent with the results of amino acids. The results indicated that the protein utilization rates of the two frying methods in vitro were similar.

## 4 CONCLUSION

The effects of TF and AFF on the quality attributes of sturgeon steak in terms of MC, color, texture, amino acids, volatile compounds, and digestibility characteristics were studied. The results revealed that along with the increase of temperature during air‐frying, the AFF sturgeon steak had crispy appearance while the moisture was well preserved inside, and the lipids were easily oxidized and degraded to volatile compounds. Results obtained from 17 kinds of amino acid illustrated that sturgeon steak after air‐frying at 160℃ for 15 min contained higher essential amino acid contents, which indicated high‐quality fried product. Compared with TF sturgeon steak, the AFF sturgeon steak presented higher MC and similar textural property, for the total digestive process, AFF sturgeon steak had the higher digestibility. Furthermore, volatile compounds composition displayed that AFF sturgeon steak produced a lot of pleasant aromas and less carcinogen. Therefore, we speculated that AFF was a more suitable method for sturgeon steak production and processing.

## CONFLICTS OF INTEREST

The authors declare there is no conflict of interest.

## AUTHOR CONTRIBUTIONS

**Li Liu:** Conceptualization (equal); Formal analysis (equal); Methodology (equal); Writing‐original draft (equal); Writing‐review & editing (equal). **Pan Huang:** Data curation (equal); Methodology (equal). **Wei Xie:** Conceptualization (equal); Writing‐review & editing (equal). **yujin li:** Funding acquisition (equal); Project administration (equal). **haiyan wang:** Funding acquisition (equal); Project administration (equal). **He Xu:** Investigation (equal); Resources (equal). **xiaodong zhou:** Funding acquisition (equal); Project administration (equal). **Yuanhui Zhao:** Funding acquisition (equal); Project administration (equal); Resources (equal); Writing‐review & editing (equal).

## ETHICAL STATEMENT

This study does not involve any human or animal testing.
